# *Origanum vulgare* ssp. *hirtum*: From Plant to 3D-Printed Gummies with Antioxidant and Anti-Inflammatory Properties

**DOI:** 10.3390/gels11040246

**Published:** 2025-03-26

**Authors:** Brayan J. Anaya, Lina Raudone, Isabel Ureña-Vacas, Amadeo Sanz-Perez, Mindaugas Marksa, Gabriele Vilkickyte, Juan José García-Rodríguez, Dolores R. Serrano, Elena González-Burgos

**Affiliations:** 1 Department of Pharmaceutics and Food Technology, School of Pharmacy, Complutense University of Madrid, 28040 Madrid, Spain; branaya@ucm.es; 2 Department of Pharmacognosy, Lithuanian University of Health Sciences, Sukileliu Av. 13, 50162 Kaunas, Lithuania; lina.raudone@lsmu.lt; 3 Laboratory of Biopharmaceutical Research, Institute of Pharmaceutical Technologies, Lithuanian University of Health Sciences, Sukileliu Av. 13, 50162 Kaunas, Lithuania; gabriele.vilkickyte@lsmu.lt; 4 Department of Basic Health Sciences, Faculty of Health Science, Rey Juan Carlos University, Av. de Atenas, s/n, 28922 Alcorcón, Madrid, Spain; isabel.urena@urjc.es; 5 Department of Pharmacology, Pharmacognosy and Botany, Faculty of Pharmacy, Complutense University of Madrid, 28040 Madrid, Spain; 6 Department of Analytical and Toxicological Chemistry, Lithuanian University of Health Sciences, Sukileliu Av. 13, 50162 Kaunas, Lithuania; mindaugas.marksa@lsmu.lt; 7 Department of Microbiology and Parasitology, Faculty of Pharmacy, Complutense University, Plaza Ramón y Cajal s/n, 28040 Madrid, Spain; jjgarc01@ucm.es; 8 Institute of Industrial Pharmacy, Complutense University of Madrid, 28040 Madrid, Spain

**Keywords:** *Origanum vulgare* ssp. *hirtum*, antioxidant, anti-inflammatory, 3D-printed, gummies

## Abstract

This study investigates the phytochemical profile, antioxidant and anti-inflammatory properties, and 3D-printing application of *Origanum vulgare* L. ssp. *hirtum* extract. The extract revealed a diverse range of phenolic compounds, with rosmarinic acid as the predominant compound (47.76%). The extract showed moderate to high lipoxygenase inhibition (IC_50_ = 32.0 µg/mL), suggesting its potential as an anti-inflammatory agent. It also exhibited strong antioxidant activity, with hydrogen peroxide scavenging (SC_50_ = 99.2 µg/mL) and hydroxyl radical scavenging (IC_50_ = 64.12 µg/mL) capabilities. In cellular studies, high concentrations (50 µg/mL and 100 µg/mL) significantly decreased intracellular ROS production in Caco-2 cells (reductions exceeding 53% and 64%, respectively). Moreover, the extract suppressed NO production in LPS-stimulated J774A.1 macrophages in a concentration-dependent manner. The study also explores the incorporation of the extract into 3D-printed gummies. The gels exhibited a shear-thinning behavior, which was essential for successful extrusion-based 3D printing. The incorporation of *Origanum* extract significantly influenced the mechanical strength and compaction properties of the 3D-printed gummies before breaking (1.6-fold increase) allowing for a better mouth feeling. PXRD and FTIR analyses confirmed the amorphous nature of the 3D-printed gummies and the interaction between active ingredients and excipients utilized for printing. These findings demonstrated the potential for semisolid extrusion 3D printing at room temperature to transform a culinary herb (*Origanum vulgare* spp. *hirtum*) into a healthcare product with antioxidant and anti-inflammatory properties.

## 1. Introduction

*Origanum vulgare* L. ssp. *hirtum* (Lamiaceae family), known as Greek oregano, is native to the Mediterranean region and found extensively in Albania, Greece, and Türkiye [1]. Greek oregano has long been, and continues to be, an essential component of the Mediterranean diet [2,3]. Beyond its culinary use as a seasoning, oregano has a rich history in traditional medicine. When consumed orally, it has been primarily utilized for its digestive properties, including carminative and antispasmodic effects, as well as its respiratory benefits as an expectorant. Applied topically, it has been used for its analgesic, antiseptic, and wound-healing properties [4].

Notably, several of these medicinal applications have been officially acknowledged by the Committee on Herbal Medicinal Products (HMPC) of the European Medicines Agency (EMA) for *Origanum majorana* L. and *Origanum dictamnus* L., which are other species within the same genus (EMA/HMPC/63479/2015, EMA/HMPC/200429/2012). These species, along with *Origanum vulgare* L., are collectively referred to as “oregano” because of their shared organoleptic properties [4].

Albania boasts a rich diversity of flora, with 10% of its plant species classified as aromatic and medicinal, including *Origanum vulgare* L. [5]. Several studies have investigated the genetic diversity and various chemotypes of these species in the region [5,6,7]. These studies primarily focus on defining chemotypes based on their essential oil composition. This essential oil is known to be rich in carvacrol and/or thymol, along with p-cymene and γ-terpinene, though many other compounds have been identified across different varieties [8]. The biological properties of *O. vulgare* have traditionally been attributed to its essential oil, which is well known for its antimicrobial activity [9], as well as antioxidant, anti-inflammatory, and anticancer properties [10,11,12,13,14,15,16]. However, the medicinal significance of its polar fraction is also noteworthy. Methanolic extracts of *Origanum vulgare* L. ssp. *hirtum* from Türkiye have been analyzed, demonstrating antioxidant and antimicrobial properties [17,18]. Furthermore, Vujicic et al. (2015) [19] reported partial protection against diabetes through antioxidant and anti-inflammatory mechanisms in an in vivo study. Published studies show differences in their phytochemical profiles, similar to those observed in essential oils, as noted by Petrakis et al. (2023) [8]. These variations underscore the need for further research in this area.

Moreover, essential oils are volatile and temperature-sensitive, leading to partial losses during cooking, drying, and storage for commercial purposes [20]. While these losses are not complete, it is important to account for the effects of other non-volatile compounds present in oregano. Due to these reasons and the scarcity of studies with extracts, we have investigated the antioxidant and anti-inflammatory properties of the extracts of the aerial parts of *Origanum vulgare* L. ssp. *hirtum*.

Three-dimensional (3D) printing technology has revolutionized personalized medicine by enabling the production of patient-specific medical devices, implants, and drug delivery systems [21,22,23]. Techniques such as Fused Deposition Modelling (FDM) and semi-solid extrusion (SSE) are commonly used for producing oral solid formulations. These methods offer high precision and versatility, making them suitable for incorporating various active pharmaceutical ingredients [24]. Combining natural extracts with 3D-printed dosage forms can improve patient acceptability and can address the solubility and absorption challenges associated with herbal extracts [25].

In recent years, this strategy has gained significant attention, particularly in the fields of bone and skin tissue engineering. For instance, ginger and garlic extracts have been incorporated into 3D-printed bone scaffolds to enhance their bioavailability, promoting osteogenesis in both in vitro studies and a rat distal femur model [26]. Similarly, 3D-printed bone scaffolds containing brucine, a compound found in the seeds of *Strychnos nux-vomica* L., have been explored for their antimicrobial properties [27]. In wound healing applications, natural extracts such as *Centella asiatica* L. Urb have been integrated into 3D-printed hydrogels, significantly accelerating wound healing and reducing inflammatory responses in an in vivo mice model [28]. Likewise, 3D-printed hydrogels incorporating *Scutellaria baicalensis* Georgi. extract have shown similar effects on human normal skin fibroblasts [29]. Other studies have explored the use of marine-derived natural compounds, such as collagen extracted from the marine sponge *Chondrilla caribensis* Rützler, Duran, and Piantoni to enhance the properties of skin dressings [30]. Beyond tissue engineering, this technology has also been applied to the development of controlled-release tablets. For example, *Mucuna pruriens* (L.) DC. extracts have been successfully incorporated into 3D-printed formulations using a semisolid extrusion technique [31].

This study aimed to evaluate the in vitro antioxidant and anti-inflammatory properties of *Origanum vulgare* L. ssp. *hirtum* extracts and develop 3D-printed loaded gummies.

## 2. Results and Discussion

### 2.1. Phytochemical Analysis

**Figure 1:**
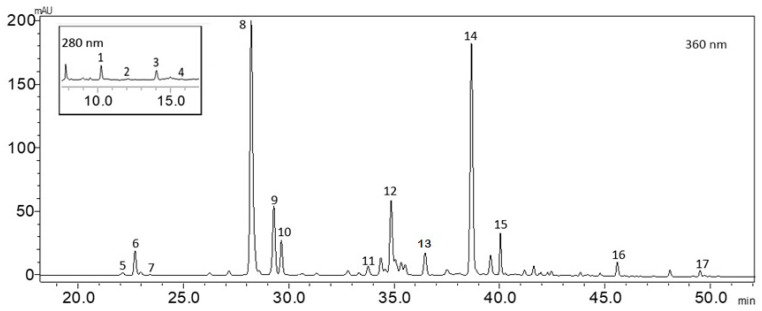
Chromatogram of HPLC analysis of the extract of the aerial parts of *Origanum vulgare* L. ssp. *hirtum.* Peak number as follows: 1—Gallocatechin; 2—Procyanidin B1; 3—Protocatechuic acid; 4—Chlorogenic acid; 5—Caffeic acid; 6—Luteolin 3,7-diglucoside; 7—Luteolin 7-rutinoside; 8—Luteolin 7-glucoside; 9—Luteolin 7-glucuronide; 10—Luteolin glycoside; 11—Apigenin 7-glucoside; 12—Luteolin glycoside II; 13—Luteolin glycoside III; 14—Rosmarinic acid; 15—Apigenin glycoside; 16—Luteolin; 17—Apigenin.

The phenolic profile of *Origanum vulgare* L. ssp. *hirtum* is a diverse complex of phenolic acids, flavonols, and flavan-3-ols (Table 1 and Figure 1). Rosmarinic acid was identified as the predominant compound, accounting for 47.76% of all detected phenolic compounds. This finding is consistent with previous studies, as species of the genus *Origanum* belonging to the subfamily *Nepetoidae* and the tribe *Mentheae* are known to be rich in rosmarinic acid, which contributes significantly to their antioxidant and anti-inflammatory properties [32]. Other identified phenolic acids, including protocatechuic acid, caffeic acid, and chlorogenic acid, were present in lower concentrations, with amounts of 1767.5 µg/g ± 88.38 µg/g, 341.50 µg/g ± 17.0 µg/g, and 22.50 µg/g ± 1.1 µg/g, respectively (Table 1). The relatively low levels of simple phenolic acids, such as caffeic acid (341.5 µg/g) and chlorogenic acid (22.5 µg/g), are consistent with findings in other *Origanum* species, where these compounds serve as precursors for more complex phenolic structures such as rosmarinic acid [33]. Nevertheless, the phytochemical data on *Origanum vulgare* L. ssp. *hirtum* remain scarce [34,35]. The complex of flavonoids is composed of a notable amount of apigenin and luteolin glycosides, particularly luteolin-7-glucoside (16,789.5 µg/g ± 839.4 µg/g), followed by luteolin-7-glucuronide (5811 µg/g) and various other luteolin glycosides (luteolin glycoside II—6711.5 µg/g), indicating a broad spectrum of luteolin derivatives, which in total account up to 30% of all the identified compounds. Additionally, apigenin derivatives, present in lower concentrations (most notably apigenin-7-glucoside at 826.0 µg/g ± 41.3 µg/g), contribute to the overall flavonoid content. The flavan-3-ols group was predominated by gallocatechin (20,544.5 µg/g ± 127.2 µg/g), which constituted 16.29% of all identified compounds. Other studies have reported additional flavonoids, such as kaempferol [35]. Moreover, Koukoulitsa et al. (2006) [34] identified other compounds namely chrysoeriol, quercetin, diosmetin, eriodictyol, cosmoside, and vicenin-2, among others. However, these compounds were not detected in our samples.

### 2.2. In Vitro Anti-Inflammatory and Antioxidant Activities

Inflammation is a biological defense mechanism aimed at eliminating damage and promoting tissue repair. However, its dysregulation and chronic nature are linked to the development of several diseases. Similarly, while the production of reactive oxygen species (ROS) plays a protective role, an imbalance between ROS and antioxidant defines can lead to oxidative stress, resulting in cellular and tissue damage. These two processes are closely interconnected: inflammatory responses trigger ROS production as a defense mechanism, which induces oxidative stress. This oxidative stress can then activate inflammatory pathways, establishing a vicious cycle that sustains chronic inflammation [36].

A common pharmacological strategy to reduce inflammation involves inhibiting proinflammatory enzymes such as cyclooxygenases (COX-1, COX-2), which are involved in the synthesis of prostaglandins; lipoxygenases (5-, 12-, 15-LOX), which are associated with leukotriene production; and xanthine oxidase (XO), which generates ROS during the conversion to uric acid, further activating proinflammatory pathways [37,38,39]. As shown in Table 2, the studies using soybean LOX-1, which mimics mammalian 15-LOX, *Origanum vulgare* L. ssp. *hirtum* extracts exhibited notable lipoxygenase inhibition, with an IC_50_ value of 32.0 µg/mL ± 2.8 µg/mL [40]. Although this value differs significantly from the standard control (naproxen: 3.4 µg/mL ± 0.4 µg/mL) when compared to the literature values for various medicinal plants, the activity of *Origanum vulgare* L. ssp. *hirtum* extracts can be classified as moderate to high [41]. Recent studies on mammalian LOX with rosmarinic acid have also shown in vitro inhibition and strong binding affinity in molecular docking models (with −7.6 kcal/mol values), indicating that hydrogen bonds and hydrophobic interactions are primarily responsible for this binding [42]. Moreover, lithospermic acid B isolated from the polar extracts of *Origanum vulgare* L. ssp. *hirtum* have shown inhibitory activity on soybean lipoxygenase with IC_50_ value of 0.1 mM [43].

On the other hand, *Origanum vulgare* L. ssp. *hirtum* extracts showed no inhibition in the XO assay. Despite this, as noted in studies by Yuk et al. (2023), glycosides exhibited weaker inhibition compared to aglycones, as glycosyl substitution interferes with binding to the XO active site [44]. As shown in Table 1, several flavonoids, such as apigenin-7-glucoside, luteolin-3,7-diglucoside, luteolin-7-glucoside, and luteolin-7-glucuronide, are present in higher concentrations than their aglycones. Therefore, following ingestion and the action of intestinal glucosidases, their in vivo effects may be enhanced.

**Figure 2:**
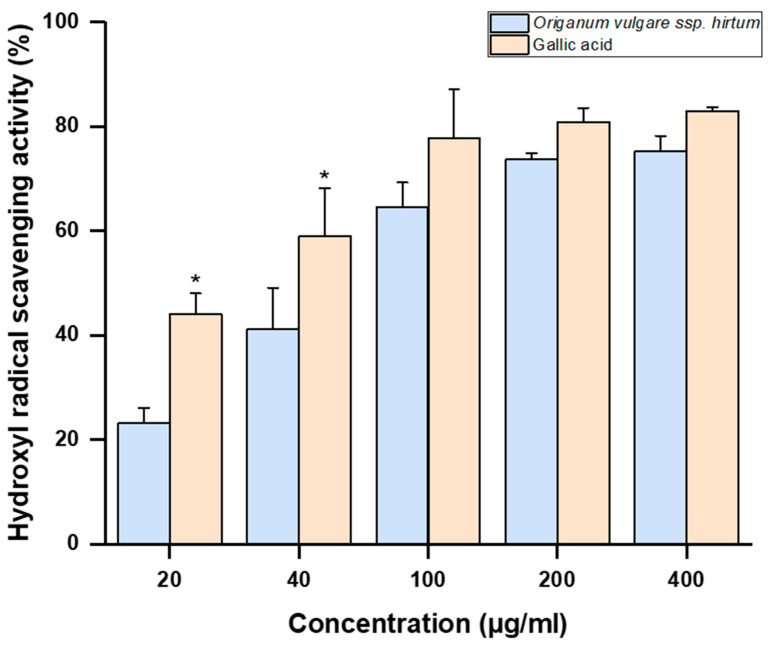
Hydroxyl radical scavenging activity (%) of the *Origanum vulgare* L. ssp *hirtum* extract. Gallic acid was used as an antioxidant reference compound. Results are expressed as means ± SD of at least three independent experiments (* *p* < 0.05 versus *Origanum vulgare* L. ssp. *hirtum*).

Another potential strategy to reduce chronic inflammation involves targeting reactive oxygen and nitrogen species (ROS/RNS), such as superoxide anion (O_2_−), hydroxyl radical (•OH), nitric oxide (NO), peroxyl radical (ROO•), and hydrogen peroxide (H_2_O_2_). The phenolic profile of *Origanum vulgare* L. ssp. *hirtum* suggests a high antioxidant capacity due to significant levels of rosmarinic acid, gallocatechin, and various luteolin glycosides [45]. As shown in Table 2, *Origanum vulgare* L. ssp. *hirtum* extracts have shown free radical scavenging activity, with hydrogen peroxide scavenging SC_50_ values of 99.2 µg/mL ± 6.8 µg/mL. This shows that oregano has a greater hydrogen peroxide scavenging capacity than other medicinal plants such as *Atalantia ceylanica* (IC_50_ = 388.11 μg/mL) and *Semecarpus parvifolia* (IC_50_ = 156.25 μg/mL) [46]. Moreover, the extract of *Origanum vulgare* L. ssp. *hirtum* exhibited hydroxyl radical scavenging activity in a concentration dependent manner (Figure 2). Gallic acid was used as antioxidant reference compound. The IC_50_ values were 64.12 µg/mL for *Origanum vulgare* L. ssp. *hirtum* and 24.73 µg/mL for gallic acid (antioxidant reference compound). Previous studies have demonstrated that the essential oils of *Origanum majorana* had hydroxyl radical scavenging activity with an IC_50_ value of 67.11 µg/mL [47]. Additionally, while XO activity was not directly inhibited, its effects could potentially be diminished through indirect mechanisms involving ROS reduction. The synergistic activity of active compounds, primarily polyphenols, present in extracts, which reduce reactive oxygen and nitrogen species levels and inhibit enzymes involved in the production of inflammatory mediators, offers a promising strategy to alleviate the proinflammatory state associated with chronic diseases and stress.

**Figure 3:**
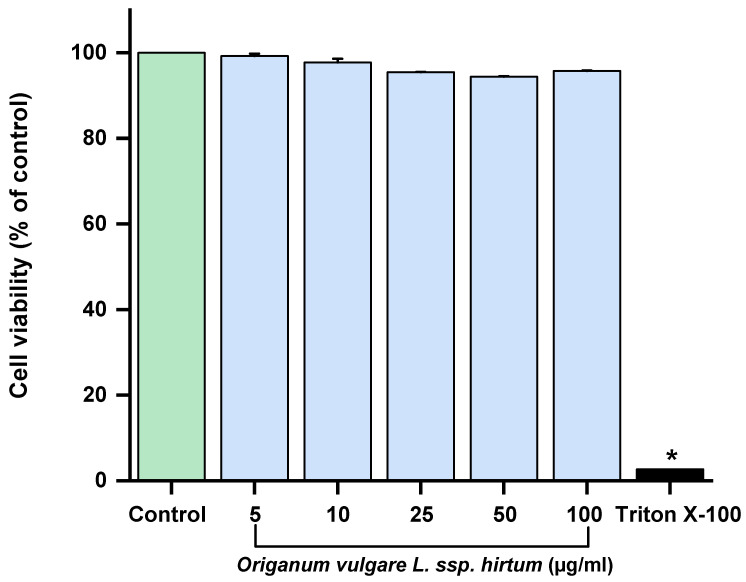
Effect of *Origanum vulgare* L. ssp. *hirtum* extract on cell viability. Cells were treated with different concentrations (5 µg/mL,10 µg/mL, 25 µg/mL, 50 µg/mL, and 100 µg/mL) of extract for 24 h. Triton was used as positive control. Cell viability was assessed using MTT assay. Results are expressed as means ± SD of at least three independent experiments (* *p* < 0.05 versus control cells).

Next, we evaluated the antioxidant and anti-inflammatory activities of the oregano extract in cellular models. Caco-2 cells are widely used as a gastrointestinal model because they mimic the properties and functions of enterocytes in the intestinal epithelium [48]. As shown in Figure 3, none of the *Origanum vulgare* L. ssp. *hirtum* extract concentrations affected the viability of Caco-2 cells even at the highest concentration (100 µg/mL) that shows antioxidant and anti-inflammatory properties.

**Figure 4:**
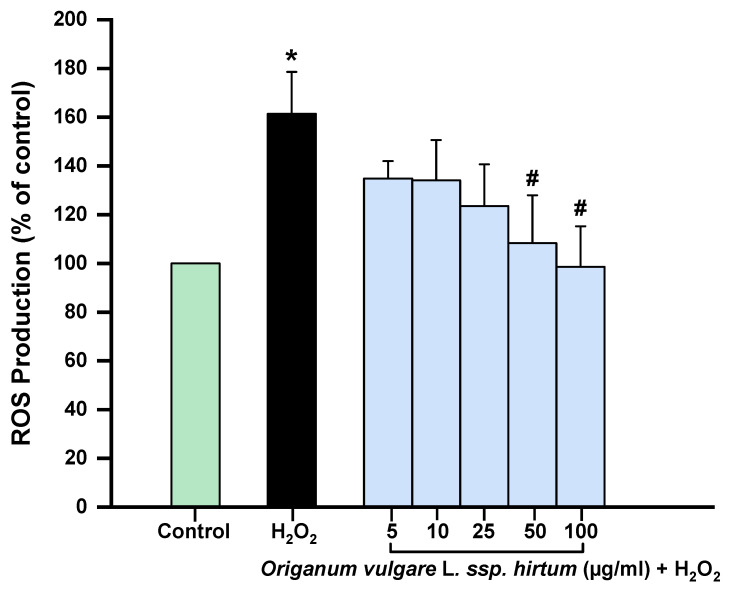
Effect of *Origanum vulgare* L. ssp. *hirtum* extract on intracellular ROS production. Cells were treated with different concentrations (5 µg/mL,10 µg/mL, 25 µg/mL, 50 µg/mL, and 100 µg/mL) of extract and with hydrogen peroxide (1 mM). Intracellular ROS production was assessed using 2′7′-dichlorofluorescein (DCF) assay at 6 h. Results are expressed as means ± SD of at least three independent experiments (* *p* < 0.05 versus control cells; # *p* < 0.05 versus hydrogen peroxide).

On the other hand, the highest concentrations of the extracts tested (50 µg/mL and 100 µg/mL) significantly decreased intracellular ROS production induced by hydrogen peroxide, with reductions exceeding 53% and 64%, respectively, compared to the hydrogen peroxide control. These results demonstrate the antioxidant capacity of oregano in an oxidative stress model (Figure 4). Similar results have been observed in previous studies published on the effects of oregano essential oil on different cell types. The protective effect against hydrogen peroxide-induced damage has been attributed, among other compounds, to carvacrol and thymol [49,50]. However, one of the limitations of essential oils is their poor solubility, high volatility, and sensitivity to UV light and heat, which represent certain disadvantages compared to extracts [51]. Moreover, regarding oregano extracts, Kubiliene et al. (2023) [51] demonstrated that the ethanolic extract of *Origanum onites* significantly reduced malondialdehyde (MDA) concentration in the liver in an in vivo mouse model induced by ethanol administration. This antioxidant effect was attributed to the compound rosmarinic acid (RA).

**Figure 5:**
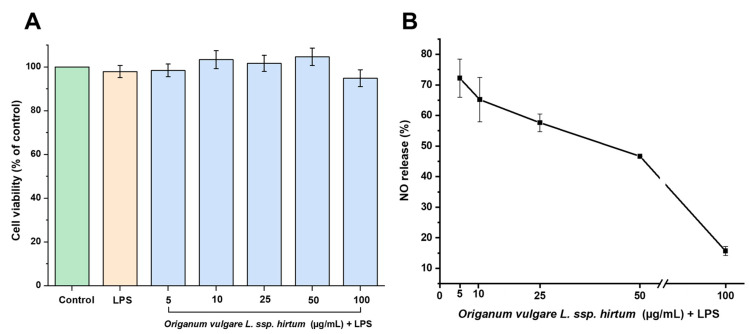
Effect of *Origanum vulgare* L. ssp. *hirtum* extract on J774A.1 cell viability and NO production. (**A**) Macrophage cells were pre-treated with oregano extract (from 5 µg/mL to 100 µg/mL for 1 h) followed by LPS treatment (1 μg/mL for 24 h). Cell viability was determined using MTT assay. (**B**) NO production was assessed using Griess reagent assay. Data are expressed as mean ± SD. Experiments were performed in triplicate.

The essential oil of *Origanum vulgare* has been recognized for its anti-inflammatory effects, which contribute to tissue remodeling and wound healing, making it valuable for dermatological applications [52]. Additionally, *Origanum* extracts exhibit strong antioxidant activity, which plays a crucial role in managing oxidative stress-related conditions [53]. *Origanum vulgare* ssp. *hirtum* extracts are particularly rich in phenolic compounds, including flavonoids such as luteolin, kaempferol, and apigenin, which are well-known for their antioxidant properties [35]. Our study supports these findings, as Figure 5B demonstrates that *Origanum vulgare* ssp. *hirtum* extract significantly suppressed NO production in LPS-stimulated cells in a concentration-dependent manner. Moreover, no cytotoxic effects were observed on J774A.1 macrophage cell viability when cells were pre-treatment with *Origanum vulgare* L. ssp. *hirtum* extract (5 µg/mL,10 µg/mL, 25 µg/mL, 50 µg/mL, and 100 µg/mL for 1 h (Figure 5A)) before LPS stimulation (1 µg/mL for 24 h).

### 2.3. 3D-Printing Gummy of Origanum vulgare *L. ssp.* hirtum Extract and Characterization

Semi-solid extrusion (SSE) is a is a commonly used technique for printing gummies [54]. The method selected for 3D printing can significantly influence the rheological and mechanical properties of gummies. Shear-thinning inks are crucial for successful extrusion-based 3D printing due to their unique rheological properties, which allow for controlled flow and material deposition. These inks exhibit a decrease in viscosity with increasing shear rate, facilitating smooth extrusion through a nozzle while maintaining structural integrity post-deposition. This behavior is essential for achieving high-resolution prints and complex geometries [55]. After extrusion, shear-thinning inks quickly recovered their viscosity, which helps preserve the structural integrity of the printed layers [56].

**Figure 6:**
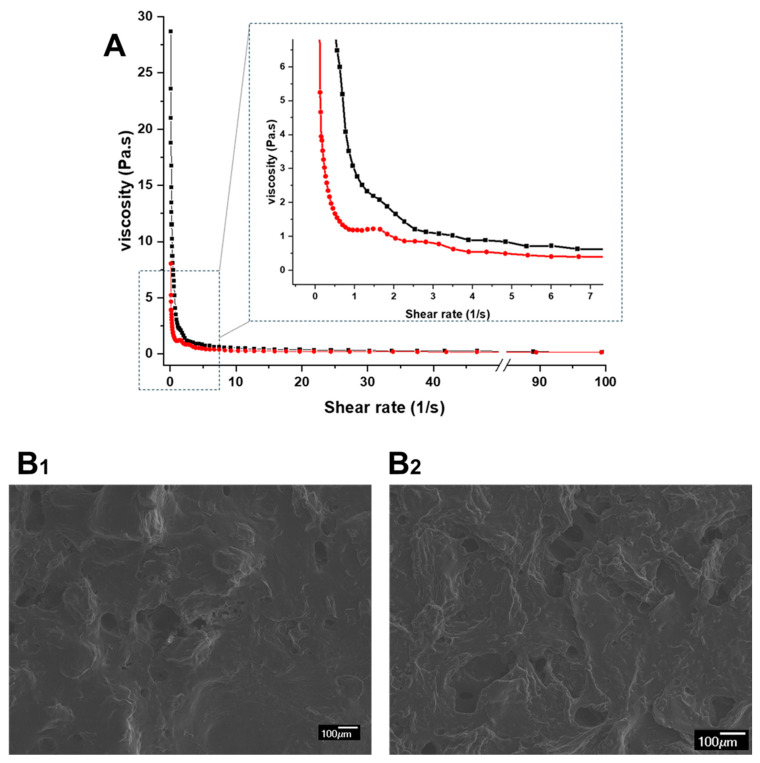
(**A**) Rheological behavior of gel used for 3D printing, and (**B1**,**B2**) SEM micrographs of 3D-printed gummies. (**A**,**B1**) Blank 3D-printed gummy without *Origanum vulgare* L. ssp. *hirtum* extract (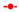, red color); (**A**,**B2**) 3D-printed gummy with *Origanum vulgare* L. ssp. *hirtum* extract (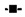, black color).

Our inks exhibited this pseudoplastic behavior, which aligns with findings reported by Santamaría et al. (2024) [57]. The incorporation of *Origanum vulgare* L. ssp. *hirtum* extract resulted in a slight increase in viscosity (Figure 6A, black color), while maintaining suitable printing parameters. However, slight differences were observed in the final 3D-printed gummy containing *Origanum* extract (34.0 ± 0.6 mm × 33.7 ± 0.4 mm and 3.6 ± 0.1 mm height with an average mass of 1733.8 ± 14.3 mg) compared to the control (33.5 ± 0.3 mm × 33.9 ± 0.2 mm and 4.0 ± 0.1 mm height with an average mass of 1747.7 ± 12.8 mg).

SEM micrographs (Figure 6(B1,B2)) revealed distinct differences in surface morphology between the blank 3D-printed gummy and the loaded one. The *Origanum* gummy (Figure 6(B2)) exhibited a more heterogenous surface compared to the control (Figure 6(B1)), likely due to incorporation of the plant extract in the ink.

The mechanical properties of 3D-printed gummies, particularly their breaking force, are crucial for ensuring their suitability for consumption, especially in pediatric/geriatric populations. Breaking force serves as an indicator of the gummies’ firmness and structural integrity, influencing both the sensory experience and the controlled release of active pharmaceutical ingredients (APIs) [57,58]. The composition of gummy formulations plays a significant role in determining their mechanical properties. In the study by Zhou et al. (2023) [59], the incorporation of peach gum polysaccharide (PGP) into gelatin-based gummies enhanced their rheological properties, resulting in a denser gel structure and improved mechanical stability. This modification not only enhanced the gummies’ printability but also increased their breaking force, making them more robust for handling and consumption.

**Figure 7:**
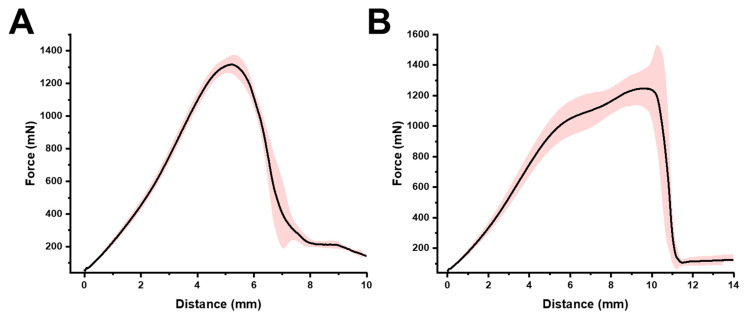
Breaking force of 3D-printed gummy. (**A**) Blank 3D-printed gummy without *Origanum vulgare* L. ssp. *hirtum* extract; (**B**) loaded 3D-printed gummy with *Origanum vulgare* L. ssp. *hirtum* extract.

In our study, the mechanical properties of the 3D-printed gummies were assessed through breaking force analysis (Figure 7). The maximum recorded force exerted by each gummy before the breaking point was 1315.6 mN ± 57.2 mN, and 1247.1 mN ± 124.2 mN for the blank 3D-printed control gummy and loaded 3D-printed gummy, respectively. Additionally, the distance travelled by the probe before the breaking probe was 6 mm for the blank 3D-printed gummy and 10 mm for the loaded one, which correlates with the area under the curve (2968.7 mN s ± 171.7 mN·s, and 4868.8 mN ± 703.2 mN·s for the blank and extract-loaded gummies respectively). These results indicate that the incorporation of *Origanum* extract significantly influenced the mechanical strength of the 3D-printed gummies, allowing for a larger compaction before breaking and hence better sensorial feeling.

The apparent contradiction between higher viscosity and greater deformation capacity can be explained by specific molecular interactions between polyphenolic compounds in the *Origanum* extract and the polymeric network of the gummy formulation [60,61]. This phenomenon occurs primarily through hydrogen bonding between the hydroxyl-rich polyphenolic compounds, particularly rosmarinic acid (47.76%) and flavonoids, and the hydroxyl groups present in gelatin and agar-agar. These interactions create a more flexible cross-linked network that can undergo greater elastic deformation before permanent structural failure. Additionally, at the molecular level, these polyphenolic compounds may function as plasticizers within the polymeric matrix, increasing polymer chain mobility while maintaining overall structural integrity [62,63,64].

This dual role of reinforcement and plasticization explains the seemingly contradictory mechanical properties we observed: the significant increase in probe travel distance before breaking (approximately 10 mm for extract-loaded gummies versus around 6 mm for the control gummy) despite higher initial viscosity. The phenolic compounds effectively modulate the cross-linking density and create a network architecture that distributes applied stress more efficiently throughout the structure. This modified network allows the material to undergo controlled and extended deformation under stress rather than immediate failure, resulting in the observed 1.6-fold increase in compressibility before breaking. These improved mechanical properties are particularly advantageous for oral formulations, providing enhanced mouthfeel and chewability while maintaining structural integrity during handling and storage.

Our study builds upon previous investigations of *Origanum* extracts while extending the application through innovative 3D printing technology. The phytochemical profile we observed aligns with findings by Michalaki et al. (2023) [35], who similarly identified rosmarinic acid as a predominant compound in *Origanum vulgare* extracts, though with regional variations in concentration. Our approach leverages the precision and flexibility of 3D printing technology, which offers advantages in developing personalized dosage forms, as demonstrated in similar applications [58].

### 2.4. Physicochemical Characterization

**Figure 8:**
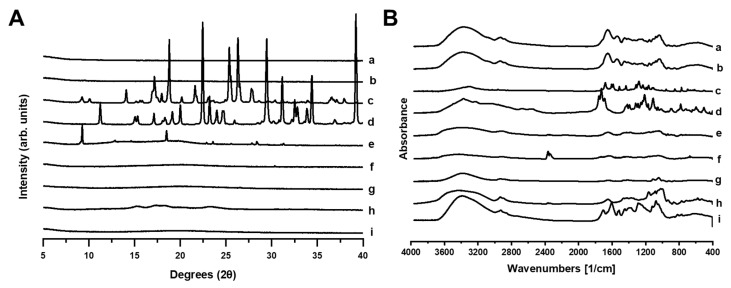
(**A**) PXRD and (**B**) FTIR analysis. (a) Loaded 3D-printed gummy with *Origanum vulgare* L. ssp. *hirtum* extract, (b) blank 3D-printed gummy without *Origanum vulgare* L. ssp. *hirtum* extract, (c) unprocessed agar–agar, (d) unprocessed gelatine, (e) unprocessed starch, (f) unprocessed citric acid, (g) unprocessed nipagin, (h) unprocessed xanthan gum, and (i) unprocessed *Origanum vulgare* L. ssp. *hirtum* extract.

PXRD analysis (Figure 8A) revealed the amorphous nature of both 3D-printed gummies with or without *Origanum vulgare* L. ssp. *hirtum* extract. The FTIR spectra (Figure 8B) confirmed the interaction of the Origanum extract compounds with the excipients of the gummy, as evidenced by characteristic shifts in peaks. In the 1600 cm^−1^–1700 cm^−1^ region, characteristic peaks associated with both C=O stretching vibrations and aromatic C=C vibrations of phenolic compounds showed notable shifts, indicating hydrogen bonding between the extract and polymeric excipients. This spectral region is particularly significant given the high content of rosmarinic acid (47.76% of all detected phenolic compounds) and flavonoids in our extract, which contains both carbonyl groups and aromatic ring structures. Additionally, the distinctive peak at approximately 2400 cm^−1^ observed in unprocessed citric acid, attributed to O-H stretching of carboxylic groups, appeared significantly diminished in the loaded gummies. This suggests interaction between the carboxyl groups of citric acid and either the hydroxyl-rich polyphenolic compounds from the *Origanum* extract or the polymeric components of the formulation, further confirming the complex molecular network formed during gummy preparation.

**Figure 9:**
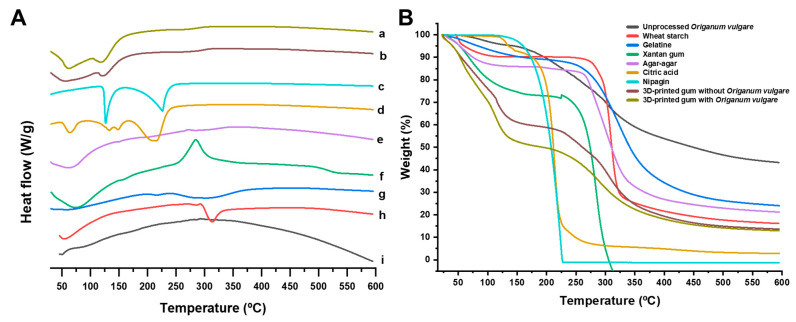
(**A**) DSC and (**B**) TGA analysis. (a) 3D-printed gummy with *Origanum vulgare* L. ssp. *hirtum* extract, (b) 3D-printed gummy without *Origanum vulgare* L. ssp. *hirtum* extract, (c) unprocessed agar–agar, (d) unprocessed gelatine, (e) unprocessed starch, (f) unprocessed citric acid, (g) unprocessed nipagin, (h) unprocessed xanthan gum, and (i) unprocessed *Origanum vulgare* L. ssp. *hirtum* extract.

DSC thermograms (Figure 9A) of the *Origanum vulgare* L. ssp. *hirtum* extract showed no endothermic events which can be attributed to its amorphous nature. However, both the blank and loaded 3D-printed gummies exhibited two endothermic events starting at 40 and 100 °C which correlated with a depression in the melting point for gelatine and agar-agar respectively. TGA curves (Figure 9B) showed a fast degradation profile for both the blank and loaded gummy attributed to the lower melting points observed in the DSC. This indicates a high sensibility to temperature and hence, the need for storage below 40 °C.

## 3. Conclusions

In conclusion, *Origanum vulgare* L. ssp. *hirtum* extract exhibits considerable potential for medical applications, primarily owing to its antioxidant and anti-inflammatory properties, which are attributed to its rich composition of phenolic compounds. The incorporation of this extract into 3D-printed gummy presents promising opportunities for pharmaceutical innovation, transforming a culinary medicinal herb into a healthcare product to enhance patient compliance and to preserve the antioxidant and anti-inflammatory properties of the plant. However, challenges remain in optimizing the formulation and manufacturing processes to ensure the stability of the active compounds over prolonged periods, efficacy, and safety. Further research is essential to fully assess the clinical applicability of *Origanum vulgare* extracts and address issues related to regulatory approval, cost-effectiveness, and scalability. Despite these challenges, the combination of *Origanum vulgare* extract and 3D printing offers a promising strategy for developing advanced therapeutic solutions, potentially enhancing the management of oxidative stress and inflammation.

## 4. Materials and Methods

### 4.1. Chemicals and Reagents

All solvents for high performance liquid chromatography (HPLC) studies were of analytical or chromatographic grade: 99.9% acetonitrile from Sigma-Aldrich (Steinheim, Germany), 99.8% trifluoroacetic acid from Merck (Darmstadt, Germany), and purified water was prepared using a Milli–Q (Millipore, Bedford, MA, USA) water purification system. The following HPLC purity standard materials were used: 3-O-caffeoylquinic acid (chlorogenic acid), 4-O-caffeoylquinic acid, luteolin, luteolin-7-O-glucoside, caffeic acid, rosmarinic acid, luteolin-7-O-rutinoside, luteolin-7-O-glucuronide, apigenin, gallocatechin, protocatechuic acid, procyanidin B1, apigenin-7-O-glucoside, vesbascoside, vanillic acid, and hesperidin from Sigma-Aldrich. The remaining reagents used in the antioxidant and anti-inflammatory activity studies were also purchased from Sigma-Aldrich. Purified water was produced using an Elix 3, Millipore water system (Merck, Madrid, Spain).

### 4.2. Plant Material

Plant material (*Origanum vulgare* ssp. *hirtum*), collected in Valbona (Albania) in August 2021, was taxonomically identified through morphologic analysis. A voucher specimen (MAF: 181692) was deposited in the Herbarium of the Faculty of Pharmacy (MAF), University Complutense of Madrid (Spain). The air-dried plant material samples were milled to homogenous powder (passing through a 355 µm sieve and kept in the dark in sealed containers until extraction) using a Retsch 200 mill (Haan, Germany).

### 4.3. Preparation of Plant Extracts

A precise weight of 20 g of milled plant material was extracted with 250 mL of 70% aqueous acetone by sonication in an Elmasonic P ultrasonic bath (Singen, Germany) for 15 min. The extraction procedures were repeated two times. Extracts were subjected to centrifugation (3000× *g*, 10 min) in a Biofuge Stratos centrifuge (Hanau, Germany) and filtered through a paper filter. The obtained extract was evaporated in an IKA RV 10 rotary evaporator (Staufen, Germany) at 40 °C until the full acetone removal and freeze-dried to dry extract powder. For the HPLC analysis, 0.01 g of dry extract (precise weight) were dissolved in 10 mL of 70% *v*/*v* methanol All obtained extracts were filtered through 0.22 µm pore size membrane filters (Carl Roth GmbH, Karlsruhe, Germany) and kept at 4 °C until further analyses.

### 4.4. HPLC-PDA and HPLC-MS Analysis

HPLC analysis was carried out using a “Waters e2695 Alliance system” (Waters, Milford, MA, USA) coupled with a Waters 2998 photodiode array detector as outlined in the method for phenolic compounds and triterpenic compounds by Raudone et al., 2017 [65]. Phenolic compounds were separated on an ACE C18 column (ACT, UK) (150 mm × 4.6 mm, 3 μm particle size) with a mobile phase consisting of eluent A (0.05% trifluoroacetic acid) and eluent B (acetonitrile). The gradient elution profile was as follows: 0 min–5 min–12% B, 5 min–50 min–12%–30% B, 50 min–51 min–30%–90% B, 51 min–56 min–90% B, 57 min–12% B. The flow rate was maintained at 0.5 mL/min, and the injection volume was 10 μL. Quantification of phenolic compounds was carried out based on linear calibration curves of external standards, with the exception of luteolin glycosides I, II, and III and apigenin glycoside, due to the unavailability of commercial standards. These compounds were tentatively quantified using calibration curves of standard substances with corresponding chemical structures.

For HPLC-MS analysis, a Shimadzu Nexera X2 LC-30AD HPLC system (Shimadzu, Tokyo, Japan) with an LCMS-2020 mass spectrometer (Shimadzu, Tokyo, Japan) was used. Chromatographic separation was performed under the same conditions as the HPLC method mentioned earlier. The electrospray ionization (ESI) parameters were optimized with an interface temperature of 350 °C, desolvation line (DL) temperature of 250 °C, heat block temperature of 400 °C, nebulizing gas flow at 1.5 L/min, and drying gas flow at 10 L/min. Both positive and negative ionization modes were employed, alternating between the two. The *m*/*z* ranges was set from 50 *m*/*z* to 2000 *m*/*z*, with a scan speed of 5000 u/s in positive ion mode and 15,000 µ/s in negative ion mode, using 0.1 *m*/*z* steps. Table 1 lists all identified compounds with their UV maximum absorbance and mass after ionization. Compound identification was performed by comparing the obtained mass spectra with literature data and structures from freely accessible databases.

### 4.5. In Vitro Anti-Inflammatory Activity

#### 4.5.1. Xanthine Oxidase (XO) Inhibition

The xanthine oxidase activity assay was conducted according to the standard method with slight modifications [66]. Positive control, allopurinol, and *Origanum vulgare* ssp. *hirtum* extracts were dissolved in dimethyl sulfoxide (DMSO). Subsequent dilutions with potassium phosphate buffer (50 mM, pH 7.4) were performed to achieve concentrations ranging from 5 µg/mL–500 µg/mL for plant extracts and 2 µg/mL–100 µg/mL for allopurinol. In a 96-well plate, a reaction mixture containing 150 µL of phosphate buffer, 10 µL of xanthine oxidase (0.1 U/mL), and 10 µL of test solution (either positive control or extracts) was preincubated at 25 °C for 15 min. Then, 30 µL of hypoxanthine (0.1 mM) was added as a substrate. Sample blanks were subtracted. The absorbance was measured at 290 nm over a 30 min period using a SPECTROstar Omega microplate reader (BMG Labtech, Ortenberg, Germany). Results were expressed as IC_50_ values.

#### 4.5.2. Lipoxygenase (LOX) Enzyme Inhibition

Following the protocol of Perera et al. (2018) [67], the cuvette method was adapted to a 96-well microplate for evaluating the LOX inhibitory activity of plant extracts. Sodium phosphate buffer (0.1 M, pH 8.0) was prepared for the assay. Samples (10 µg/mL–500 µg/mL) and naproxen (2 µg/mL–50 µg/mL), used as a positive control, were dissolved in the buffer. Sample and naproxen stocks were dissolved in buffer to final concentrations ranging from 5 µg/mL–200 µg/mL (5, 10, 25, 50, 100, 200 µg/mL) and 2 µg/mL–50 µg/mL (2, 5, 10, 25, 50 µg/mL), respectively. Each well received 10 µL of sample, 110 µL of buffer, and 30 µL of enzyme (2000 U/mL), and the mixture was incubated for 10 min at room temperature. Linolenic acid, dissolved in methanol (1mM), was then added as a substrate to achieve a final volume of 150 µL. Absorbance measurements were immediately detected using a microplate reader at 234 nm for 10 min (SPECTROstar Omega microplate reader (BMG Labtech, Ortenberg, Germany)). The inhibition rate was calculated according to the established formula and expressed as IC_50_ values.

### 4.6. In Vitro Antioxidant Activity

#### 4.6.1. Hydrogen Peroxide (H_2_O_2_) Scavenging

The hydrogen peroxide scavenging activity of the samples was evaluated using the colorimetric assay described by Fernando et al. (2015) [46]. Plant samples were dissolved in sodium phosphate buffer (84 mM, pH 7.0) with a minimal amount of DMSO. Dilutions were prepared in the same buffer at different concentrations (10 µg/mL–500 µg/mL). Gallic acid was used as a standard (2.5 µg/mL–20 µg/mL). The reaction mixture consisted of 80 µL H_2_O_2_ (0.7mM) and 50 µL sample/standard/solvent, which was incubated for 3 min at room temperature. This was followed by 80 µL phenol (12 mM), 25 µL 4-aminoantipyriene (0.5 mM) and 15 µL horseradish (HR) peroxidase (1.0 U/mL). After 30 min incubation at 37 °C, the absorbance was measured at 504 nm using a SPECTROstar Omega microplate reader (BMG Labtech, Ortenberg, Germany). For each sample concentration, a sample blank was evaluated, and the background was subtracted. SC_50_ values were calculated.

#### 4.6.2. Hydroxyl Radical Scavenging

Hydroxyl radical-scavenging activity was determined using a 2-deoxyribose oxidative degradation oxidation assay with some modifications [68]. Plant extracts were incubated with 2-deoxy-D-ribose (10.4 mM), FeCl_3_ (50 μM), H_2_O_2_ (10 mM), ascorbic acid (1.0 mM), and with EDTA (52 µM) in KH_2_PO_4_/KOH buffer (30 mM, pH 7.4) at 37 °C for 60 min. The positive control consisted of the H_2_O_2_/Fe^3+^/ascorbic acid system mixture without the extract. Following incubation, a butylated hydroxytoluene solution (2%) and 2-thiobarbituric acid (1%) in trichloroacetic acid were added. Then, the samples were heated in a water bath at 85 °C for 20 min. The reaction was stopped in an ice-water bath for 5 min. MDA-TBA (thiobarbituric acid) products were extracted with n-butanol. Absorbance was measured at 532 nm using a microplate reader (SpectraSTAR Nano, BMG Labtech, Madrid, Spain).

The hydroxyl radical scavenging capacity was assessed by measuring the percentage inhibition of 2-deoxy-D-ribose oxidation induced by hydroxyl radicals. The hydroxyl radical scavenging activity (%) was calculated according to Equation (1):

(1) $$\%\;\mathrm{hydroxyl}\;\mathrm{radical}\;\mathrm{scavenging}\;\mathrm{activity}=\frac{A_{0}-A_{1}}{A_{0}}\times 100$$

where *A*_0_ is the absorbance of the positive control and *A*_1_ is the absorbance of the extract tested. The IC_50_ values (the concentration of extract in mL required to inhibit 50% of the degradation of 2-deoxy-D-ribose) were subsequently calculated.

### 4.7. Cell Assays

#### 4.7.1. Cell Culture

The human colorectal cancer cell lines (Caco-2 cells, HTB-37, kindly provided by Mª Auxiliadora Dea Ayuela) were grown in Dulbecco’s modified Eagle’s medium (DMEM) supplemented with 10% FBS and 1% penicillin/streptomycin. The murine macrophage J774A.1 cells (ATCC^®^ TIB-67™, kindly provided by Mª Auxiliadora Dea Ayuela) were grown in RPMI-1640 medium supplemented with 10% FBS and 1% penicillin/streptomycin. Both cell lines were incubated at 37 °C in 95% humidified air with 5% CO_2_.

#### 4.7.2. Cell Treatments

Caco-2 cells were treated with 5 µg/mL, 10 µg/mL, 25 µg/mL, 50 µg/mL, and 100 µg/mL concentrations of oregano extracts for 24 h. Additionally, hydrogen peroxide (300 µM) was administered to test the protective potential on intracellular ROS production.

J774A.1 cells were treated with 5 µg/mL, 10 µg/mL, 25 µg/mL, 50 µg/mL, and 100 µg/mL concentrations of oregano extracts for 1 h, then treated with lipopolysaccharide (LPS) at 1 μg/mL for 24 h for cell viability and nitric oxide (NO) assays.

#### 4.7.3. Cell Viability Assay

Cytotoxic activity was performed using the MTT method [69]. Caco-2 cells and J774A.1 cells were treated with different concentrations (5 µg/mL, 10 µg/mL, 25 µg/mL, 50 µg/mL, and 100 µg/mL) of oregano extracts for 24 h. After treatments, cells were exposed to MTT solution (5 mg/mL for 2 h). Formazan crystals were subsequently dissolved using a DMSO reagent. Absorbance was measured at 550 nm with a SPECTROstar BMG microplate reader (BMG LABTECH, Ortenberg, Germany). Cell viability was calculated as a percentage relative to untreated control.

#### 4.7.4. Intracellular Reactive Oxygen Species (ROS) Production

The measurement of intracellular ROS production was assessed using 2′,7′-dichlorofluorescein diacetate assay [70]. Caco-2 cells were exposed to DCFDA (20 µM, 30 min) in the dark. Fluorescence intensity was measured at an excitation/emission wavelength of 485/528 nm using a microplate reader (FLUOstar OPTIMA, BMG Labtech, Ortenberg, Germany). Intracellular ROS production was calculated as a percentage relative to untreated control.

#### 4.7.5. Nitric Oxide Determination

After treatments, cell culture supernatants of J774A.1 cells were mixed with Griess reagent (2% sulphanilamide + 2% naphthyl ethylenediamine dihydrochloride in 10% H_3_PO_4_) at room temperature. Absorbance was measured at 550 nm with a SPECTROstar BMG microplate reader (BMG LABTECH, Ortenberg, Germany) [71].

### 4.8. 3D-Printing Gummy Formulation with Origanum Vulgare

#### 4.8.1. Composition of Gummy Formulation

*Origanum vulgare* ssp. *hirtum* extract. Xanthan gum was purchased from MP Biomedicals (Madrid, Spain). Wheat starch was purchased from Guinama (Madrid, Spain). Citric acid monohydrate (99.5% purity) was purchased from Thermo Scientific (Madrid, Spain). Nipagin (methyl paraben) was purchased from Fagron (Madrid, Spain). Gelatin (Royal Mondeléz, Madrid, Spain), glycerin (Azucren, Artynnova reposteria Sevilla), agar-agar (Vahiné, McCormik, Madrid, Spain), liquid sweetener (Steviat, SoriaNatural, Madrid, Spain), and lemon essential oil (Herbolario Navarro, Madrid, Spain) were bought from a local store (Madrid, Spain).

#### 4.8.2. 3D-Gummy Preparation

The methodology for 3D printing of gummy formulation was adapted from [57]. The composition comprised *Origanum vulgare* ssp. *hirtum* extract (20.4% *w*/*v*), gelatin (49.0% *w*/*w*), starch (16.3% *w*/*w*), agar-agar (8.2% *w*/*w*), xanthan gum (4.1% *w*/*w*), citric acid (1.6% *w*/*w*), and nipagin (0.4% *w*/*w*), supplemented with lemon essential oil (3 drops), glycerin (3 drops), and sweetening agent (2 drops), all dissolved in purified water (4 mL) (Table 3). The lemon essential oil and sweetening agent were specifically incorporated to mask the intense flavor of the *Origanum vulgare* L. ssp. hirtum extract, thereby improving palatability and enhancing patient acceptability of the final formulation. This formulation was utilized for the preparation of both blank and extract-loaded gummies.

The printing ink was prepared through sequential incorporation of components. Initially, *Origanum vulgare* ssp. *hirtum* extract, citric acid, and nipagin were dissolved in heated purified water (2 mL, 50 °C) under gentle, continuous agitation for approximately 2 min. Starch was subsequently incorporated until a homogeneous dispersion was achieved, followed by the addition of the remaining water (2 mL). The gelling agents (gelatin, xanthan gum, and agar-agar) were then individually introduced with sufficient hydration time allowed between additions. Finally, lemon essential oil, sweetening agent, and glycerin were incorporated to complete the formulation. The prepared ink was transferred to 5 cc plastic syringes fitted with dispensing tips (diameter: 0.58 mm) for loading into the REG4LIFE 3D bioprinter (REGEMAT 3D, Granada, Spain).

The three-dimensional model, designed using Regemat Software, consisted of a cube (34 mm × 34 mm × 4 mm) with a layer height of 0.40 mm. The printing process was conducted at room temperature with an extrusion rate of 2.00 mm/s, employing a solid infill pattern with a 90° orientation angle. The entire printing procedure was completed in approximately 2 min.

#### 4.8.3. Rheology Analysis

Rheological characterization of the formulated inks was performed in triplicate using an AR2000 Rheometer (TA Instruments, Newcastle, DE, USA) equipped with a 40 mm flat plate geometry. The rheological behavior was assessed by measuring viscosity as a function of shear rate. The experimental protocol involved a continuous increase in shear rate at 0.33 Pa/s up to a maximum of 100 s^−1^, while maintaining a constant temperature of 40 °C. The resultant rheological data were subsequently processed and analyzed using TA Universal Analysis software v5.5.24. (Waters, New Castle, DE, USA).

#### 4.8.4. Scanning Electron Microscopy

The morphological profile of both blank (gummies without *Origanum vulgare* ssp. *hirtum* extract) and extract-incorporated 3D-printed gummies were comprehensively evaluated. For examination purposes, sections were obtained from the central region of each gummy formulation and carefully positioned on 32 mm specimen stubs, allowing complete desiccation prior to analytical assessment. The prepared samples underwent gold sputter coating for a duration of 180 s using a Q150RS QUORUM Metallizer (Madrid, Spain). Morphological characterization was conducted using a JSM-IT700HR scanning electron microscope (JEOL, Tokyo, Japan) operated at an accelerating voltage of 10 kV.

#### 4.8.5. Mechanical Strength

The mechanical integrity of the 3D-printed gummies was quantitatively assessed by determining breaking force parameters using a 5 mm diameter spherical stainless-steel ball probe with appropriate adapter attachment. Each gummy sample was precisely positioned within the film support apparatus, and the measurement involved a pre-test probe advancement rate of 0.5 mm/s, followed by a test velocity of 1 mm/s, and concluding with a post-test retraction rate of 10 mm/s. The penetration depth was standardized to 15 mm, with force application initiated at a trigger threshold of 5 N. Key mechanical parameters, maximum force (mN), probe displacement distance (mm), and force-displacement integral (mN·mm), were continuously recorded at a high-resolution data acquisition rate of 200 points per second. The resultant mechanical profiles were comprehensively analyzed using Exponent software (version 8.0.14.0) [72].

#### 4.8.6. Physicochemical Characterization

Powder X-Ray Diffraction (pXRD) analysis was performed using a Philips^®^ X’Pert-MPD X-ray diffractometer (Malvern Panalytical^®^; Almelo, The Netherlands) equipped with Ni-filtered Cu Kα radiation (1.54 Å). The diffraction patterns were recorded at operational parameters of 40 kV voltage and 40 mA current. Data acquisition was conducted at a step scan rate of 0.05°/s across a 2-theta range of 5° to 40° [73].

Fourier Transform Infrared (FTIR) spectroscopic analysis was conducted using a Nicolet Nexus 670–870 system (Thermofisher, Madrid, Spain). Spectral data were collected across the wavelength range of 400 cm^−1^ to 4000 cm^−1^ with a resolution of 1 nm per step. The resulting spectral data were processed and interpreted using Spectragryph software (version 1.2.9, Oberstdorf, Germany), with appropriate normalization procedures applied to facilitate comparative analysis.

Thermal characterization was performed through Differential Scanning Calorimetry (DSC) coupled with Thermogravimetric Analysis (TGA) using an SDT Q600 instrument (TA Instruments, Elstree, UK). Standard thermal scans were conducted on powder samples (5–6 mg) under nitrogen atmosphere. The thermal program consisted of a constant heating rate of 10 °C/min from 24 °C to 600 °C. The calorimetric system was calibrated using indium as the reference standard. Glass transition temperatures were determined as the midpoint of the thermal transition and reported as the mean of triplicate measurements (*n* = 3).

### 4.9. Statistical Analysis

The results are expressed as the mean ± standard deviation (SD) of three independent experiments. Statistical analysis was conducted using SigmaPlot 11.0, with analysis of variance (ANOVA) followed by Tukey’s post hoc test. A *p*-value of <0.05 was considered statistically significant. The data were plotted using Origin Pro 2021 versión 9.8 (OriginLab Corporation, Northampton, MA, USA).

## Figures and Tables

**Table 1:** Chemical composition of extract of *Origanum vulgare* L. ssp. *hirtum* and -MS (negative ionization mode) data of identified phenolic compounds.

Peak No.	Tentative Identity	Rt (min)	λ max	(M-H)^−^ (*m*/*z*)	Other Ions^−^ (*m*/*z*)	Amount, µg/g
1	Gallocatechin *	10.29	280	305	289	20,544.5 ± 1027.2
2	Procyanidin B1 *	11.89	280	577	425–407–289	1348.5 ± 67.4
3	Protocatechuic acid *	14.03	219–259–293	153	108	1767.5 ± 88.4
4	Chlorogenic acid *	15.37	330	353	191	22.5 ± 1.1
5	Caffeic acid *	22.14	323	179	135	341.5 ± 17.1
6	Luteolin 3,7-diglucoside *	22.71	253–347	609	447–285–248	2546.5 ± 127.3
7	Luteolin 7-rutinoside *	23.42	254–347	593	285	167.0 ± 8.3
8	Luteolin 7-glucoside *	28.26	254–347	447	285	16,789.5 ± 439.5
9	Luteolin 7-glucuronide *	29.29	253–346	461	285–248	5811.0 ± 290.6
10	Luteolin glycoside I **	29.63	253–346	579	285–417–248	1839.0 ± 91.9
11	Apigenin 7-glucoside *	33.79	266–332	431	269	826.0 ± 41.3
12	Luteolin glycoside II **	34.84	253–346	417	285–248	6711.5 ± 335.6
13	Luteolin glycoside III **	36.52	251–346	475	285–248	1774.5 ± 88.7
14	Rosmarinic acid *	38.72	329	359	161	60,241.0 ± 3012.1
15	Apigenin glycoside **	40.05	266–337	635	269–117	3199.5 ± 159.9
16	Luteolin *	45.58	250–346	285	248	2133.5 ± 106.6
17	Apigenin *	49.76	266–335	269	149–117	71.5 ± 3.6

* Identification of these compounds made by comparison with standards. ** were tentatively quantified using calibration curves of substances with similar chemical structure; RT: retention time; λ max—UV maximum; *m*/*z*—mass-to-charge ratio.

**Table 2:** Antioxidant and anti-inflammatory activities of extract of *Origanum vulgare* L. ssp. *hirtum.* Results are presented as mean values ± SD (*n* = 3, *p* < 0.05). SC_50_: Concentration for 50% free radical scavenging. IC_50_: Concentration for 50% enzyme inhibition.

	Antioxidant Properties (SC_50_ µg/mL)	Anti-Inflammatory Properties (IC_50_ µg/mL)	
	Hydroxy Peroxide Scavenging	Xanthine Oxidase Inhibition	Lipoxygenase Inhibition
Oregano extract	99.2 ± 6.8	>500	32.0 ± 2.8
	Reference compounds		
Gallic acid	5.6 ±0.46	-	-
Allopurinol	-	17.2 ± 0.8	-
Naproxen	-	-	3.4 ± 0.4

**Table 3:** Composition of 3D-printing gel formulation.

**Component**	**Concentration (% *w*/*w*)**	**Amount (mg)**
*Origanum vulgare* ssp. *hirtum* extract	20.4	250
Gelatin	49.0	600
Starch	16.3	200
Agar-agar	8.2	100
Xanthan gum	4.1	50
Citric acid	1.6	20
Nipagin	0.4	5
**Additional components**		
Lemon essential oil *		3 drops
Glycerin		3 drops
Sweetening agent *		2 drops
Purified water		4 mL

* These components were added to mask the intense flavor of the *Origanum* extract.

## Data Availability

The original contributions presented in this study are included in the article. Further inquiries can be directed to the corresponding authors.
